# The Bloody Crossroads: Interactions between Periodontitis and Hematologic Diseases

**DOI:** 10.3390/ijms25116115

**Published:** 2024-06-01

**Authors:** Michał Łobacz, Paulina Mertowska, Sebastian Mertowski, Aleksandra Kozińska, Wojciech Kwaśniewski, Marek Kos, Ewelina Grywalska, Mansur Rahnama-Hezavah

**Affiliations:** 1 Chair and Department of Oral Surgery, Medical University of Lublin, 20-093 Lublin, Poland; michal.lobacz@umlub.pl (M.Ł.); mansur.rahnama-hezavah@umlub.pl (M.R.-H.); 2 Department of Experimental Immunology, Medical University of Lublin, 20-093 Lublin, Poland; paulinamertowska@umlub.pl (P.M.);; 3 Student Research Group of Experimental Immunology, Medical University of Lublin, 20-093 Lublin, Poland; 4 Department of Gynecologic Oncology and Gynecology, Medical University of Lublin, Staszica 16 Street, 20-081 Lublin, Poland; wojciech.kwasniewski@umlub.pl; 5 Department of Public Health, Medical University of Lublin, 20-400 Lublin, Poland; marekkos@umlub.pl

**Keywords:** periodontitis, hematologic diseases, leukemia, multiple myeloma, anemia, microorganisms

## Abstract

Periodontitis is a common oral condition that can have a significant impact on the overall health of the body. In recent years, attention has been paid to potential relationships between periodontitis and various hematological disorders. This publication aims to present information available in the literature on this relationship, focusing on examples of red blood cell disorders (such as aplastic anemia and sickle cell anemia) and white blood cell disorders (such as cyclic neutropenia, maladaptive trained immunity, clonal hematopoiesis, leukemia, and multiple myeloma). Understanding these associations can help physicians and dentists better diagnose, monitor, and treat patients associated with both groups of conditions, highlighting the need for interdisciplinary care for patients with oral disorders and hematologic diseases.

## 1. Introduction

Periodontitis is described as a clinical condition characterized by chronic inflammation of the periodontium, which may result in the loss of periodontal ligaments and damage to the alveolar bone. Advanced stages of periodontitis can lead to tooth loss [1,2] The oral cavity is a habitat of diverse microflora, including approximately 700 different species of bacteria. The oral microbiota is a significant source of periodontitis, in which bacterial pathogens trigger an inflammatory response that damages connective tissue. The Gram-negative bacteria that cause periodontitis include *Aggregatibacter actinomycetemcomitans*, *Porphyromonas gingivalis*, *Prevotella intermedia*, and *Tannerella forsythia*. In particular, *Porphyromonas gingivalis* is the main pathogen found in most subgingival plaques in cases of chronic periodontitis [3,4]. The development of periodontitis results from an imbalance in the oral microflora and host immunity, called dysbiosis, and is one of the most common oral inflammatory conditions. It is associated with bacterial plaque, and the risk factors include poor oral hygiene, smoking, genetic predisposition, and other systemic diseases such as diabetes or hematological diseases [2,4]. How can periodontal disease be related to hematologic diseases? Many mechanisms may lead to this association. First, periodontal disease can lead to inflammation in the body, which in turn can affect the hematopoietic system. Gingivitis and periodontitis lead to excessive release of pro-inflammatory cytokines and inflammatory mediators into the bloodstream, which can trigger an immune system response and oxidative stress. This inflammation can affect the production of blood cells, including leukocytes and platelets, which can lead to hematological disorders. Secondly, there is a connection between some periodontal diseases and blood diseases such as anemia, leukemia, and multiple myeloma [5,6,7]. Additionally, the presence of bacteria in the oral cavity can lead to infections and inflammations that can spread to the bloodstream and affect the condition of the hematopoietic system. This means that bacteria that infect the gingiva can enter the bloodstream during everyday activities such as brushing teeth or using dental floss, which in turn can lead to blood infections (bacteremia) and hematological complications. As a result, there is a noticeable association between periodontal disease and hematologic diseases, which requires attention from physicians and dentists when diagnosing and treating patients [8]. Long-term treatment of periodontitis leads to significant reductions in blood inflammatory markers in patients with generalized aggressive periodontitis. These findings support the ongoing management of periodontitis to maintain systemic health [9].Therefore, this publication aims to present the information available in the literature regarding the relationship between periodontitis and hematological disorders using the example of red blood cell disorders (aplastic anemia and sickle cell anemia) and white blood cell disorders (cyclic neutropenia, maladaptive trained immunity, clonal hematopoiesis, leukemia, and multiple myeloma).

## 2. Current Classification of Periodontal Diseases

Knowledge about periodontal disease has advanced significantly, particularly with the introduction of the new classification system established at the 2017 World Workshop. This system categorizes periodontal disease based on a multidimensional grading framework, offering a more detailed approach to diagnosis and treatment [10]. Staging refers to the severity and extent of the disease, taking into account the amount of tissue lost through periodontitis and tooth loss. Its purpose is to present both the severity of the disease when it occurs and the complexity of treating the disease (American Dental Association, ADA). In this context, particular attention should be paid in this respect to periodontal diseases directly related to hematological diseases, which have been classified as manifestations of systemic diseases such as leukemia, aplastic anemia, and cyclic neutropenia. On the other hand, proposed staging provides insight into the biological characteristics of the disease, the risk of progression, the expected response to standard therapies, and the impact on systemic health. The inclusion of evidence-based risk factors such as smoking, diabetes, and genetic predispositions in the new classification system allows for a comprehensive patient assessment. This holistic approach facilitates discussion about lifestyle changes that can mitigate the progression of periodontal disease [11,12]. Periodontal treatment aims to remove biofilm and tartar using non-surgical and surgical methods, adapted to the specific stage and degree of the patient’s disease. Non-surgical procedures, such as scaling and root planing, are often the first line of defense and can be supplemented with systemic or topical antimicrobial therapies when necessary [13].

In moderate to advanced cases, surgical intervention may be necessary to reduce pocket depth and allow the patient to effectively control plaque, thereby increasing the likelihood of maintaining periodontal health. After treatment, patients enter a maintenance phase, which is crucial for monitoring periodontal stability and preventing recurrence. The frequency of maintenance visits may vary, but the literature suggests that intervals of 3–6 months may be beneficial [14]. To prevent mismanagement and potential litigation, it is necessary to communicate the diagnosis and treatment plan to the patient, ensuring that all clinical decisions are well documented and follow the standard guidelines set out in the new classification system [15].

The European Federation of Periodontology (EFP) provides extensive resources to dentists and patients. These include the Journal of Clinical Periodontology, JCP Digest, and Perio Insight, which provide expertise in periodontology and clinical practice.

## 3. The Relationship between Hematological Disorders and Periodontitis

**Figure 1:**
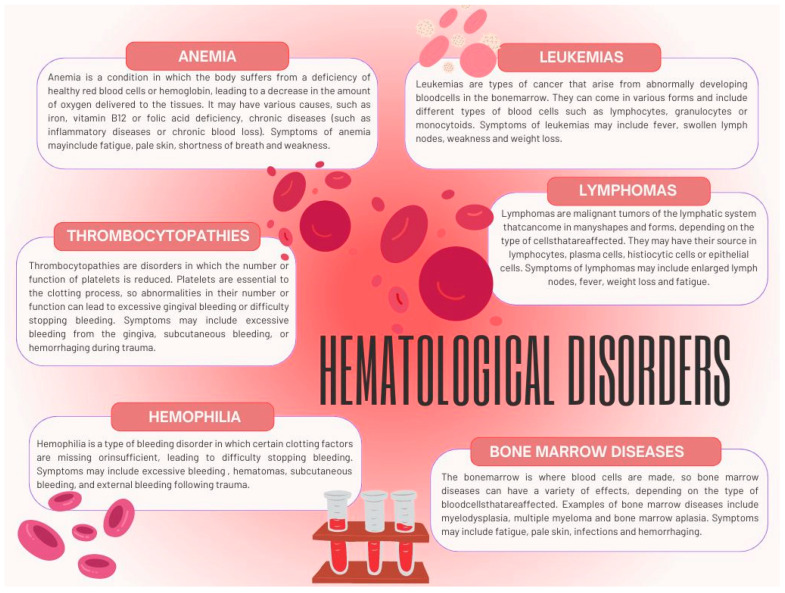
Examples of the most common hematological disorders with their characteristics, based on [16].

Hematological disorders are a group of diseases that affect the hematopoietic system, i.e., the process of formation and functioning of blood cells. They may involve various blood components, such as red blood cells (erythrocytes), white blood cells (leukocytes), platelets (thrombocytes), and plasma components. The most common hematological disorders include anemia, leukemia, lymphomas, thrombocytopathies, bone marrow diseases such as myelodysplasia or myeloma, and hemophilia (Figure 1).

**Figure 2:**
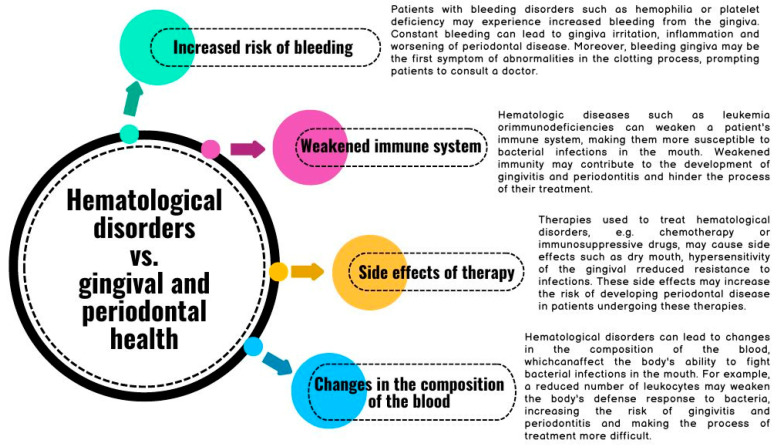
Examples of mechanisms by which hematological disorders may affect the health of the gingiva and periodontium based on [17,18,19,20].

Hematologic disorders can impact oral health, including the development and severity of periodontal diseases such as gingivitis and periodontitis. There are several mechanisms by which hematologic disorders may affect gingival and periodontal health; these are outlined in Figure 2.

The last mechanism discussed in Figure 2, regarding changes in the composition of patients’ blood, seems to be particularly important. The analysis of blood count parameters in patients with periodontal disease shows a number of significant changes, which have become the subject of several studies in recent years. Severe chronic periodontitis is associated with higher white blood cell counts, mainly in the context of neutrophils and lymphocytes. This is particularly evident in the outcomes of patients with severe disease compared to moderate disease [5]. However, as a result of non-surgical treatment, a decrease in the number of white blood cells is observed [21]. In the course of periodontitis, hypochromic microcytic anemia occurs, manifesting itself in reduced red blood cell count, hemoglobin (HGB), hematocrit (HCT), and Mean Corpuscular Volume (MCV). Additionally, increased red cell distribution width (RDW) values and erythrocyte sedimentation rates (ESR) were observed [6]. Moreover, platelets (PLTs) have a lower average volume as compared to levels in healthy volunteers constituting the control group [21]. These changes reflect the body’s inflammatory response to periodontal infection, which is the inducer of inflammation. Additionally, according to researchers, these observed changes may result from the presence of an inflammatory infiltrate, rich in leukocytes, that can enter the systemic circulation from the site of the primary infection. The constant impact of inflammation and the pathogens that cause it can stimulate the bone marrow to produce more cells that participate in the inflammatory response. Bacteria inhabiting the periodontium can also penetrate the periodontal tissue through a damaged epithelium and cause a systemic inflammatory reaction [5].

Recent research has illuminated the intricate links between systemic health and periodontal disease, highlighting the special concern for patients with hematologic disorders. Periodontitis-related bacteremia and systemic inflammation are known to exacerbate underlying diseases, including those affecting the bone marrow and hematopoietic stem cells (HSCs). The dissemination of periodontal bacteria through the bloodstream and the resulting systemic inflammatory response—characterized by elevated serum levels of interleukin-6 (IL-6) and C-reactive protein (CRP)—can have a profound impact on hematopoietic processes. For example, periodontitis is associated with altered red blood cell indices, such as decreased hemoglobin and hematocrit levels, suggesting an association with the inflammatory anemia commonly observed in chronic inflammatory conditions [16]. Moreover, the immunomodulatory effects of periodontitis on HSCs suggest that periodontal pathogens and the inflammatory environment they induce may significantly influence the differentiation and function of these cells [22].

The analysis by Hu et al. [23] revealed significant demographic and hematological differences between groups, indicating how stages of periodontitis are related to changes in hematological parameters and potential contribute to an understanding of the impact of periodontitis on overall health. This comprehensive data set allows for a detailed analysis of the progression of periodontitis and its relationship with hematologic parameters, providing valuable information for both clinical practice and further research on periodontal disease [23].

### 3.1. Diseases Related to Red Blood Cell Disorders and Periodontitis

#### 3.1.1. Aplastic Anemia (AA)

Aplastic anemia is a rare disease defined as pancytopenia with bone marrow hypocellularity without abnormal infiltrates and without increased reticulocyte concentration. Most cases are acquired and have an immunological basis, but hereditary forms also occur. Environmental factors include drugs, viruses, and toxins, but most cases are idiopathic. It may also be caused by genetic factors and inflammatory processes [24]. A reduced number of erythrocytes, leukocytes, and platelets may be observed in the peripheral blood count. These changes result from the inability of the marrow to produce blood and result in pancytopenia [25]. Multiple pro-inflammatory cytokines, including interferon-γ (IFN-γ), tumor necrosis factor-α (TNF-α), IL-8, IL-12p70, IL-17, IL-23, and IL-27, as well as thrombopoietin and granulocyte colony-stimulating factor, occur in increased concentrations in AA, which may explain the coexistence of inflammation. Damage to the bone marrow by the immune system leads to increased apoptosis of progenitor cells. However, the exact pathogenesis of acquired AA is still unclear. In the primary form, the deficiency of HSC is responsible for the observed changes in bone marrow cellularity [26].

Modern literature data show the commonness of oral lesions in the course of AA, which are directly related to pancytopenia. These include petechial lesions, swelling, spontaneous gingival bleeding, ulceration, pallor of the gingiva, and severe periodontal disease. Cases of advanced or rapidly progressive periodontitis have been reported, accompanied by long-term neutropenia, and may be caused by qualitative defects in neutrophils, including impaired leukocyte adhesion. Thrombocytopenia is associated with coagulation disorders, which may manifest in bleeding gingiva and hemorrhagic ecchymoses [27]. A method of treating the form of AA associated with excessive activation of the immune system is immunosuppressive therapy with cyclosporine A. One of its side effects may be impaired immunity and, consequently, periodontitis [28]. As shown by the clinical case of a 36-year-old woman, reduced body immunity resulting from neutropenia in the course of aplastic anemia is associated with the risk of developing severe periodontitis with severe alveolar bone resorption [29]. Adults over 18 years with anemia are more likely to develop chronic periodontitis compared to those without anemia. This systematic review and meta-analysis highlights the need for comprehensive periodontal care in anemic patients to mitigate this risk [30].

#### 3.1.2. Sickle Cell Disease (SCD)

Sickle cell anemia is the most common form of hemoglobinopathy, an inherited group of blood diseases [31]. As a result of inheriting two abnormal copies of the β-globin (HBB) gene located on chromosome 11, abnormal hemoglobin S is produced, resulting in the formation of stiff, sickle-shaped erythrocytes [32]. Characteristic symptoms of this disease include anemia, swelling of the hands and feet, bacterial infections, and stroke. Pain attacks known as sickle cell crises may develop [31]. Treatment for SCD includes preventing infection by receiving vaccinations and antibiotics, drinking plenty of fluids, folic acid supplementation, and symptomatic treatment with pain medications. Other treatment methods include blood transfusions and hydroxyurea therapy, or in some cases, bone marrow cell transplantation [31,33]. In 2023, new gene therapies were approved [34]. The peripheral blood count shows decreased levels of HGB, erythrocytes, and HCT, with a simultaneously high level of reticulocytes, resulting from the marrow’s compensation for the destruction of sickled blood cells. The blood smear may show features of hyposplenism (thyroid cells and Howell–Jolly bodies) [35].

Patients with sickle cell disease, as a result of their immune deficiency, are more susceptible to infections, including periodontal disease, which is associated with systemic inflammation [36]. There is evidence showing that oral infections can worsen the general condition of patients with sickle cell disease [37]. These patients have a higher incidence of periodontal inflammatory disease, which is associated with elevated acute phase biomarkers, compared to healthy controls [38].

#### 3.1.3. Anemia of Chronic Disease (ACD)

Anemia of chronic disease is a form of anemia associated with chronic infections, chronic activation of the immune system, and malignant tumors. All of these entities cause increased levels of interleukin-6, which stimulates the production and release of hepcidin from the liver. This results in the blocking of ferroportin—a protein responsible for the transport of iron from the intestines and iron-storing cells (e.g., macrophages). This results in a reduction in the level of iron, necessary for the synthesis of hemoglobin [39]. It is usually mild, but in some cases, it may be severe. In most cases, it is a type of normocytic anemia, but it can also be microcytic. Due to the similar picture of peripheral blood morphology with iron deficiency anemia (microcytic, hypochromic), distinguishing one form from the other is often difficult. In ACD without iron deficiency, ferritin concentrations are normal or high, reflecting the fact that iron is sequestered within the cells and ferritin is produced as an acute phase reactant. In iron deficiency anemia (IDA), ferritin is low. Total iron binding capacity (TIBC) is high in IDA, reflecting the production of more transferrin to increase iron binding. TIBC is low or normal in the setting of anemia associated with chronic inflammation [40].

Chronic periodontitis is a chronic inflammatory disease characterized by persistent inflammation [41]. It is speculated that low-grade systemic inflammation accompanying periodontal inflammation may cause erythropoiesis disorders and, consequently, a lower number of erythrocytes and lower HGB concentration [42,43]. Despite these speculations, however, conflicting results have been reported regarding the relationship between periodontal disease and anemia [43,44].

### 3.2. Diseases Related to White Blood Cell Disorders and Periodontitis

#### 3.2.1. Cyclic Neutropenia (CyN)

CyN is a rare hematological disorder that is one of the forms of congenital neutropenia. It usually appears about every three weeks and lasts for a few days. It results from changes in the rate of neutrophil production in the bone marrow, which causes a temporary state of low absolute number, which increases the risk of inflammation and infection. It responds well to treatment with a granulocyte colony-stimulating factor (filgastrim), which increases the number of neutrophils, shortens the length of cycles, and reduces the severity and frequency of infections [45,46]. Typically, it manifests with recurrent fever, malaise, inflammation, and bacterial infections of the respiratory tract, digestive tract, and skin, abdominal pain, and inflammation of the oral cavity [45,47]. CyN is caused by an autosomal dominant mutation in the *ELANE* gene located on the short arm of chromosome 19 (19p13.3). It encodes neutrophil elastase. This mutation results in reduced neutrophil production or excessive neutrophil apoptosis, which results in a deficiency of mature neutrophils in the blood [45,48]. The diagnosis is usually made by monitoring the absolute neutrophil count (ANC) three times a week for at least six weeks. In a morphological examination of peripheral blood, it can be observed that the absolute number of neutrophils is less than 200–500 cells/µL and is accompanied by an increase in the number of monocytes and mild quantitative disturbances of other cells, including mild anemia [47,49,50]. Oral symptoms of cyclic neutropenia include oral ulcers, angular cheilitis, periodontitis (including its severe form) with advanced tissue destruction, and premature tooth loss. These symptoms resemble those of recurrent aphthous stomatitis and aggressive periodontitis, which makes their differential diagnosis difficult, which is only possible after further hematological tests [51].

#### 3.2.2. Maladaptive Trained Immunity

Trained innate immunity (TII) is a state of increased immune reactivity based on innate immune (epigenetic) memory. It is currently believed that innate immune cells retain heterologous memory of microbial or inflammatory factors with which the body has previously been in contact. This allows for a stronger immune reaction when contact is made with them again [52]. Experimental studies show that TII may protect against infections and cancer but may be harmful, and therefore maladaptive, in chronic inflammatory diseases [53]. There is data confirming that TII leads to increased myelopoiesis and, consequently, to the production of mature myeloid cells with increased pro-inflammatory capacity [54]. TII protects the body against re-infection (with the same pathogen that caused TII in the first place), as well as against unrelated pathogens that the body is dealing with for the first time. Maladaptive TII may also promote aberrant immune responses that have the potential to exacerbate immune-mediated pathologies, thereby contributing to the prevalence and severity of chronic inflammatory diseases. Maladaptive TII can be caused by infections, purified microbial particles (in the form of vaccines), and an obesogenic diet [54].

Periodontal diseases are an example of a dysregulated balance between the local subgingival microbiome and the host immune response that is induced in response to contact with a pathogen. In patients with periodontitis, the inflammatory response not only fails to mediate protective immunity but also drives the selective expansion of pathogenic species, leading to dysbiosis. The development of microorganisms associated with inflammation increases the intensity of the inflammatory destruction process, which results in the occurrence of chronic periodontal inflammation. Systemic inflammation associated with chronic inflammatory diseases such as periodontitis may result in inappropriate recruitment of hematopoietic progenitor cells, resulting in increased myelopoiesis and the production of inflammatory myeloid cells that may inhabit distant organs and tissues, including the periodontium, where they may exacerbate local inflammation [54]. Patients with periodontitis show higher levels of pro-inflammatory cytokines such as IL-1, IL-6, TNF, IFN-γ, IL-17, oncostatin M and CRP, and fibrinogen, as well as an increased number of neutrophils compared to the healthy control group. The levels of these cytokines have been observed to decrease after successful non-surgical periodontal treatment. Bone marrow self-renewing HSCs have the ability to produce all types of mature blood cells. They have receptors for Toll-like receptors (TLRs) and growth factors and cytokines (e.g., receptors for IL-1β, IL-6) and thus may respond to systemic inflammation (or infection) by initiating increased myelopoiesis, especially of the macrophage, granulocyte, monocyte, and neutrophil lineage. Under the influence of inflammation, hematopoietic stem and progenitor cells (HSPCs) may also undergo long-term increased proliferation and skewed differentiation towards the myeloid lineage. This persistent inflammatory modulation of HSPCs, which is observed in the course of periodontitis, underlies the induction and maintenance of TII [54].

#### 3.2.3. Clonal Hematopoiesis of Indeterminate Potential (CHIP)

Clonal hematopoiesis of indeterminate potential is an aging phenomenon in which HSCs or other early blood progenitor cells differentiate into a genetically distinct subpopulation of blood cells [55]. HSPCs develop one or more somatic mutations, which are often associated with myeloid malignancies. As a result of these mutations, the mutant HSPC acquires a kind of survival/proliferation advantage, which thus enables its clonal expansion and the production of mutant daughter cells, constituting a distinct fraction of leukocytes in the peripheral blood [54,56]. Clonal hematopoiesis can occur in completely healthy people, but it has been found also in people with hematological diseases [56,57]. The incidence of clonal hematopoiesis has been found to increase with age, and it may be a precancerous lesion preceding various hematologic malignancies, such as chronic lymphocytic leukemia (CLL), hairy cell leukemia (HCL), and acute myeloid leukemia (AML) [58,59,60,61]. There are currently no therapies to slow or target the mutations underlying CHIP [55,56]. People with CHIP do not have clinical symptoms of hematological disorders, for example, cytopenias, lines displaced by a mutant cell clone. CHIP, by leading to the production and release of hyperinflammatory myeloid cells, contributes to inflammatory aging and increases systemic inflammation. This may contribute to the pathogenesis of additional inflammatory diseases, thereby increasing the susceptibility to periodontitis [54].

#### 3.2.4. Multiple Myeloma (MM)

MM is a cancer characterized by the neoplastic proliferation of plasma cells that produce monoclonal immunoglobulin. These cells multiply in the bone marrow, leading to extensive skeletal destruction, including osteolytic lesions, osteopenia, and pathological fractures [62]. MM accounts for approximately 1–2% of all cancers and over 17% of hematological malignancies, affecting mainly men and people over 60 years of age [63]. The annual incidence in North America is approximately 7 per 100,000 inhabitants, with a similar incidence observed in Europe [64,65,66]. Risk factors include increased body mass index, exposure to certain toxins, and chronic ionizing radiation, although the exact etiology remains unclear [67]. A significant risk factor is having a first-degree relative with MM, which increases the risk by approximately 3.7 times [68].

The disease typically results from the transformation and proliferation of plasma cells, often leading to the overproduction of dysfunctional immunoglobulin. This may manifest itself as the presence of M protein in serum and/or urine, often leading to increased protein concentration in serum or proteinuria [68]. MM is diagnosed based on criteria established by the International Myeloma Working Group, which include the presence of clonal plasma cells in the bone marrow and damage to a specific organ or system.

MM is a heterogeneous disease with variable progression and response to treatment. The Revised International Staging System (R-ISS) divides MM into three stages based on specific criteria, including beta-2 microglobulin levels, serum albumin concentration, serum lactate dehydrogenase (LDH) concentration, and genetic abnormalities detected by FISH. Treatment includes intensive therapy with a high risk of recurrence, including corticosteroids, immunomodulatory drugs, monoclonal antibodies, and proteasome inhibitors. Hematopoietic stem cell autotransplantation is considered for eligible patients younger than 70 years of age. Radiotherapy and bisphosphonates are used to treat bone complications [69,70]. The treatment and management of MM require close monitoring due to various potential complications, including risk of infection related to immune system dysfunction and physical factors.

Oral symptoms in MM can be significant, although they are less common. Patients may experience oral symptoms such as jaw or tooth pain, paresthesia, swelling, soft tissue tumors, tooth mobility, bleeding, and pathological fractures due to bone destruction. Approximately 14% of patients with MM experience dental symptoms, but this number may be underestimated [71]. These symptoms may mimic typical dental pathologies such as periapical or periodontal abscess, severe gingivitis, or periodontitis. Patients often report sensory disturbances, especially paresthesia of the oral cavity and surrounding areas. While classical biopsy remains the gold standard in the diagnosis of suspicious lesions in the oral cavity, fine needle aspiration biopsy may be a real alternative due to its ease, low complication rate, and rapid diagnosis [72]. Careful examination and monitoring of the oral cavity is extremely important, especially in patients with a history of MM, because changes in the oral cavity may indicate recurrence of the disease. Dental complications may also result from MM treatment, e.g., immunosuppression leading to increased caries and periodontal disease, and bisphosphonate therapy, which may cause osteonecrosis of the jaw, which may be difficult to distinguish from MM-related oral pathologies [73].

Among these oral symptoms, tongue swelling is particularly rare. This symptom may result from direct tumor infiltration of the tongue by malignant plasma cells, leading to noticeable enlargement and discomfort. Such symptoms can easily be misinterpreted as indicating milder tongue conditions, making early recognition and diagnosis difficult [69,70,74,75].

Another noteworthy symptom is osteolytic changes in the jaws. These changes result from the destructive effect of myeloma cells on bone tissue, which may manifest in the jaw area as local pain, swelling, and even pathological fractures. Although osteolytic lesions are a hallmark of MM, their occurrence in the jaw bones highlights the need for dentists to remain vigilant when dealing with patients presenting with unexplained bone pain or radiographic abnormalities in the craniofacial region [76].

Oral amyloidosis is a less common but significant symptom characterized by the deposition of amyloid proteins in oral tissues. This can lead to the formation of lumps or plaques, especially on the tongue, contributing to a feeling of stiffness or visible enlargement. Oral amyloidosis in patients with MM is a direct consequence of abnormal protein production by malignant plasma cells and serves as a key diagnostic clue [77,78].

Oral ulcers and submucosal hematomas, although seen less frequently, highlight the differential impact of MM on oral health. These symptoms may be directly attributable to the primary malignancy or may result from treatment complications, presenting unique challenges in differential diagnosis and treatment [71,79].

The rare oral manifestations of MM, including tongue swelling, osteolytic lesions, oral amyloidosis, oral ulcers, and submucosal hematomas, highlight the integral role of the oral cavity in reflecting systemic disease processes. Their diagnosis and appropriate treatment require a multidisciplinary approach, which emphasizes the importance of cooperation between hematologists, oncologists, dentists, and other healthcare professionals. This synergy is crucial not only for the accurate diagnosis and treatment of MM but also for the comprehensive care of affected patients, highlighting the need for increased awareness and expertise in oral and systemic health [71].

Common systemic manifestations of MM include osteolytic lesions, bone fractures, hypercalcemia, anemia, renal failure, and infections due to immunosuppression. Oral symptoms in patients with MM may occur in up to 14% of cases and manifest as lytic lesions in the jaws, swelling, tooth mobility, and migration due to root resorption, gingival hemorrhage, and amyloid deposits. Although primary gastrointestinal hemorrhage is a common occurrence in patients with malignant hematological diseases, it is rare in patients with MM [80]. In the field of hematologic malignancies, MM presents a unique set of challenges, primarily including oral and facial symptoms ranging from dental pain and paresthesia to gingival hemorrhage, tooth mobility, and ulceration. Despite their occurrence, these symptoms are often overlooked due to their variability and the difficulty in definitively assigning them to MM based on the clinical picture alone [81].

Osteolytic lesions, a more direct indicator of MM, occur less frequently in the oral cavity—they are found only in approximately 5.18% of patients, with a predilection for the posterior section of the mandible. This distribution is probably due to the increased hematopoietic activity in this area. Diagnostic criteria for symptomatic MM require the presence of plasmacytoma or clonal plasma cells on bone marrow biopsy, M protein in urine or serum, and evidence of organ or tissue damage associated with plasma cell proliferation [82]. To ensure prompt and accurate treatment, it is important to maintain a high index of suspicion for MM in patients with such oral symptoms, especially in the context of a known diagnosis of MM [83].

#### 3.2.5. Acute Lymphoblastic Leukemia/Lymphoma (ALL/LBL)

ALL/LBL, a malignancy of the lymphatic system affecting both children and adults, is characterized by a spectrum of symptoms resulting from abnormal proliferation of lymphocytes. In general, ALL/LBL is associated with a large and rapidly increasing number of leukocytes, especially in the case of the T lineage. In cases of bone marrow infiltration, leukopenia may also occur. Typical hematologic findings include anemia, neutropenia, and thrombocytopenia. Peripheral blood smear shows the presence of lymphoblasts, while bone marrow examination shows the dominance of blast cells with the regression of other lines [84,85,86,87]. Immunophenotyping of blood and bone marrow cells is used to determine the subtype of the disease and detect the presence of markers that may be affected by immunotherapy. Cytogenetic and molecular tests assess chromosome number and structural changes that correlate with clinical features and are used in prognostic assessment [84,85,86,87].

Symptoms in the oral cavity are particularly dangerous and require careful dental and medical treatment. Mucosal inflammation characterized by erythema, ulceration, and subsequent pain and dryness often interferes with nutrition and hydration, leading to further systemic disorders [88,89].

Salivary gland dysfunction manifesting as xerostomia [88,90,91,92] exacerbates these challenges by altering saliva’s consistency and reducing its protective properties, which are necessary to maintain oral health. This change predisposes individuals to a more frequent occurrence of opportunistic infections, such as candidiasis [88,93], and increased acidity in the oral cavity favors a cariogenic environment, increasing the risk of dental caries [88,89].

Gingivitis and gingival bleeding, often exacerbated by chemotherapy-induced thrombocytopenia, may progress to chronic periodontal disease if not treated appropriately [88,94,95,96]. Complications such as trismusn, a debilitating limitation in mouth opening, may result from fibrosis due to radiation or the direct effects of chemotherapy [88,97]. Furthermore, osteoradionecrosis remains a serious problem, especially after tooth extractions or other oral surgical procedures [88,98,99].

The clinical course of ALL/LBL, characterized by rapid progression, highlights the need for rapid and effective intervention [84,85,86,87]. Modern treatment models have significantly improved prognosis, including precision medicine and targeted therapies such as tyrosine kinase inhibitors (TKIs), monoclonal antibodies, and CAR-T cell therapy [100,101]. However, these methods pose new oral health risks, including dysgeusia and oral dysesthesia, which may further complicate patient care. [88,102]

Oral manifestations and ALL are well documented in the literature. A systematic review of studies shows that oral symptoms in patients with ALL are quite common and may result from direct toxicity of chemotherapy drugs via systemic circulation or salivary secretion, leading to oral exposure. The most common oral lesions found in children with ALL undergoing chemotherapy were mucositis, candidiasis, periodontitis, and gingivitis, and the most commonly affected sites were the oral and labial mucosa [103,104].

The interaction between leukemia and the oral microbiome exacerbates periodontal disease. The infiltration of leukemic cells into the gingiva, combined with an impaired immune response, creates conditions for the destruction of the periodontium and complicates the maintenance of oral hygiene, increasing the risk of infection [105,106].

Research suggests that markers of systemic inflammation and peripheral blood counts in patients with hematologic malignancies may predict oral health outcomes. The occurrence of gingiva and periodontal diseases in these patients correlates with their systemic condition, emphasizing the need for comprehensive oral care [107,108].

Effective treatment includes preventive dental care and a multidisciplinary approach that includes dental evaluation before and during treatment for hematologic malignancies. Preventive dental care, tailored to a patient’s specific needs, can alleviate oral health complications and improve quality of life [109,110].

Oral symptoms in patients with hematologic malignancies highlight the complex relationship between oral health and overall body health. Early detection and prevention and a multidisciplinary approach to treatment are of paramount importance in the management of these patients, highlighting the key role of dentists in the broader healthcare team [103,110].

Hematopoietic Stem Cell Transplantation (HSCT) is a procedure that replaces damaged stem cells to treat cancers like leukemia and lymphoma. A review of studies from 2000–2020 assessed HSCT’s impact on oral health, focusing on caries, periodontal conditions, and tooth loss [111,112]. The results showed no significant progression in these conditions post-HSCT, but prevalence varied widely, suggesting a need for consistent oral care. Caries and periodontitis were more common in HSCT survivors than in the general population, while tooth loss rates were similar. The evidence for all conclusions is very uncertain, indicating a need for further research. Short-term HSCT may have little to no effect on caries, periodontal conditions, and tooth loss, but the long-term impacts require more investigation to develop targeted prevention and treatment strategies. Additionally, these findings highlight the necessity for international standards of care and consistent oral health monitoring post-HSCT [113,114].

## 4. Molecular Mechanisms Linking Periodontitis and Hematologic Diseases

Recent research has provided deeper insights into the molecular mechanisms that link periodontitis with hematologic diseases. A study published in the Journal of Proteome Research [115] highlights the role of proteomic changes in patients with periodontal disease and their systemic effects. This research emphasizes how proteins involved in inflammation, immune response, and tissue regeneration are differentially expressed in periodontal disease, potentially impacting hematologic health.

Proteomic analysis has revealed that chronic periodontitis induces systemic inflammation characterized by elevated levels of pro-inflammatory cytokines such as interleukin-6 (IL-6) and C-reactive protein (CRP). These inflammatory markers can affect hematopoiesis, leading to alterations in blood cell production and function, which are critical in patients with hematologic disorders like anemia and leukemia.

The study discusses how periodontal pathogens can enter the bloodstream, especially during routine activities like brushing or flossing, leading to bacteremia. This systemic spread of bacteria and their inflammatory byproducts can exacerbate conditions such as cyclic neutropenia and multiple myeloma by further compromising the immune system and promoting chronic inflammation.

## 5. Interdisciplinary Care for Patients with Hematologic Diseases and Periodontitis

The management of patients with hematologic diseases who also suffer from periodontitis necessitates a coordinated interdisciplinary approach to ensure comprehensive care. Hematologic diseases, such as anemia, leukemia, and neutropenia, can significantly impact periodontal health, and conversely, severe periodontitis can exacerbate systemic conditions and complicate the management of hematologic diseases. Effective collaboration between dentists and hematologists is essential to address the complex needs of these patients.

Experimental induction of periodontitis using bacterial strains isolated from the human oral microbiome in rats has provided insights into disease mechanisms. These findings could pave the way for new therapeutic approaches in periodontal disease management [116].

Hematologic disorders can compromise the immune system, making patients more susceptible to infections, including periodontal infections. This bidirectional relationship underscores the need for a comprehensive treatment plan that integrates dental and medical care. Dentists must perform thorough periodontal evaluations and document any signs of periodontal disease, while hematologists should provide detailed medical histories, including the type and status of the hematologic disease, current treatments, and any potential complications from dental procedures.

The treatment of periodontitis in patients with hematologic conditions requires special considerations. Given the increased risk of infections and bleeding, periodontal treatments should be carefully planned and executed. Minimally invasive procedures are preferred to reduce the risk of complications. Regular follow-ups are crucial to monitor the patient’s periodontal health and adjust treatment plans as necessary, with continuous communication between the dentist and hematologist being vital to promptly address any changes in the patient’s health status.

Subgingival instrumentation has a positive impact on reducing systemic inflammation and serum bone resorption markers in premenopausal women with periodontitis. This prospective study emphasizes the systemic benefits of periodontal treatment in this demographic [117].

Antibiotics as an adjuvant to subgingival instrumentation significantly reduce systemic inflammation in periodontitis patients. This randomized clinical trial supports the combined use of antibiotics for enhanced periodontal treatment outcomes [118]

Advanced regenerative procedures are particularly valuable in managing periodontitis, especially for patients with systemic conditions like diabetes, which can hinder periodontal healing. The use of biomaterials in periodontal regeneration offers promising alternatives to traditional methods.

Platelet-rich fibrin (PRF) and hyperacute serum (HAS) are quantitatively associated with improved glycemic control in chronic periodontitis patients. These results highlight the potential benefits of PRF and HAS in managing periodontal disease in diabetic patients [119].

The study by Bianchi et al. [120] highlights the effectiveness of using different deproteinized bovine bone mineral (DBBM) grafts treated with low-temperature protocols. These materials promote cell proliferation and enhance the healing process by maintaining the integrity of the bone structure. The low-temperature treatment helps preserve the biological properties of the grafts, which in turn supports better integration and regeneration of periodontal tissues.

Another significant study by Mizutani et al. [121] demonstrated that periodontal regenerative therapy using an enamel matrix derivative in patients with type 2 diabetes yielded promising results under a minimally invasive surgical technique over a three-year observation period. This indicates that even in diabetic conditions, successful periodontal regeneration is achievable with the right materials and techniques. The study’s findings suggest that using minimally invasive methods can significantly improve outcomes for diabetic patients undergoing periodontal therapy.

Doxycycline and IL-17 play significant roles in enhancing the regenerative potential of periodontal ligament stem cells, which has promising implications for periodontitis treatment. These findings underscore the therapeutic potential of targeting inflammatory pathways in periodontal disease management [122].

The work of Nanayakkara et al. [123] underscores the importance of tailored dental care for individuals with complex or rare inherited bleeding disorders. The authors emphasize specific protocols for managing periodontitis non-surgically to the mitigate risks associated with invasive procedures. Their research advocates for less invasive treatments that minimize bleeding and infection risks, which is critical for patients with bleeding disorders.

Integrating these advanced regenerative procedures into the treatment plans for patients with hematologic diseases can significantly enhance the management of periodontitis. The use of biomaterials such as DBBM, combined with minimally invasive techniques, offers a viable alternative to traditional periodontal surgery. This approach not only reduces the risk of complications but also promotes faster and more effective healing of periodontal tissues, even in patients with compromised systemic health.

Oxidative stress is implicated in the hepatic inflammatory response to apical periodontitis, particularly in hyperlipidaemic rats. This study suggests that managing oxidative stress could be crucial in treating periodontal-related systemic inflammation [124].

The comprehensive care strategy should involve regular communication and coordinated efforts between dentists and hematologists to ensure that all aspects of the patient’s health are considered. This integrated approach will help in achieving better health outcomes, improving the quality of life for patients with hematologic diseases and periodontitis.

The effective management of patients with hematologic diseases and periodontitis requires an interdisciplinary approach that includes thorough assessment, coordinated treatment planning, and continuous communication between healthcare providers. Incorporating advanced regenerative procedures and adapting treatment protocols to meet the specific needs of these patients can significantly improve their overall health outcomes. This collaboration ensures that the complexities of both periodontal and hematologic conditions are adequately addressed, leading to better patient care and quality of life.

## 6. Conclusions and Clinical and Future Perspective

Based on a synthesis of oral health challenges in patients with hematologic malignancies, the conclusions highlight the interconnected nature of systemic disease and oral health. First, oral manifestations, especially periodontal disease and gingivitis, serve as critical indicators of underlying hematologic conditions and treatment side effects. Second, the compromised immune status of these patients significantly increases the risk of oral infections, requiring vigilant oral hygiene and preventive care. Third, interdisciplinary collaboration among health care providers is essential for early recognition and treatment of oral health problems, improving overall patient outcomes. Fourth, regular dental visits should be incorporated into the care plan for patients with hematologic malignancies to proactively prevent and alleviate oral health complications. Finally, further research is needed to develop targeted strategies to manage oral health complications in this vulnerable patient population, emphasizing the importance of oral hygiene in the comprehensive management of hematologic malignancies.

Periodontitis is significantly associated with an increased risk of hematologic cancer, as demonstrated in a cohort study in Taiwan. These findings suggest that periodontal health may play a crucial role in cancer prevention strategies [125].

Patients with hematologic disorders are at increased risk of developing oral health problems, including periodontal disease, due to reduced immunity. This susceptibility may result from the hematologic disease itself or be a consequence of treatment such as chemotherapy or bone marrow suppression. These treatments can significantly weaken the immune system, making it harder for the body to fight infections, including those that affect oral health. Furthermore, hematologic disorders can affect the presence of specific microorganisms in the oral cavity, potentially exacerbating conditions such as periodontal disease.

Pregnant women with chronic periodontitis exhibit higher levels of inflammatory markers compared to those without the condition. This study underscores the importance of periodontal health monitoring in pregnancy to prevent systemic inflammation [126]

Given the complexity and differential impact of hematologic disorders on oral health, especially periodontal disease, a comprehensive understanding requires examination of both the direct and indirect effects of these systemic diseases on the oral microbiota, immune response, and tissue integrity. Hematologic disorders, including anemias, leukemias, and lymphomas, can significantly alter the host immune system, making the oral cavity more susceptible to periodontal pathogens and exacerbating the severity of periodontal disease. An altered immune response may lead to a reduction in the body’s ability to fight infections, including those caused by periodontal pathogens. Additionally, treatments for hematologic disorders, such as chemotherapy, can further worsen oral health by reducing salivary secretion, leading to dry mouth (xerostomia), which exacerbates periodontal disease by providing a favorable environment for bacterial growth. Moreover, specific hematologic conditions, such as neutropenia, may directly impair the body’s defense against periodontal pathogens, increasing the risk of severe periodontal infections.

The association between periodontitis and hematologic disorders is not merely one of increased risk or severity; this is a two-way interaction in which periodontal disease may influence the course and risk of complications of hematological diseases. For example, the systemic inflammation associated with periodontitis may affect blood cell counts and function, potentially influencing the treatment and prognosis of hematologic diseases.

Asymptomatic apical periodontitis lesions are linked to a systemic inflammatory burden, as indicated by a prospective clinical study. This highlights the need for proactive dental care to reduce systemic inflammation [127].

Recent research suggests a link between periodontal disease and an increased risk of developing certain types of cancer, including hematological cancers. This association emphasizes the importance of maintaining oral health in the prevention and treatment of systemic diseases, including diseases of the hematopoietic system. Therefore, dentists must remain vigilant in managing periodontal health in patients with hematologic disorders, advocating for an integrated approach to care that addresses both oral and systemic health challenges.

There is a notable association between hematologic parameters and the severity of COVID-19 in patients with periodontitis. This case–control study emphasizes the importance of periodontal health in managing COVID-19 outcomes [128].

Recent studies identified significant associations between periodontitis and serum levels of PECAM-1 and TRIM21, suggesting these proteins as novel markers of inflammation in periodontitis. These findings underscore the potential of PECAM-1 and TRIM21 to serve as biomarkers for the systemic inflammation caused by periodontal disease. Understanding these biomarkers is crucial, as they provide insight into the molecular mechanisms linking periodontitis with systemic health conditions, including hematologic disorders.

Given the systemic implications of periodontitis, maintaining periodontal health is essential for preventing and managing systemic inflammation and its effects on hematologic health. This highlights the importance of interdisciplinary collaboration between dental and medical professionals to ensure comprehensive care for patients, addressing both oral and systemic health issues [115]

## Data Availability

No new data were created or analyzed in this study.

## Abbreviations

HSCT	Hematopoietic Stem Cell Transplantation
IL-6	Interleukin-6
CRP	C-reactive protein
ADA	American Dental Association
EFP	European Federation of Periodontology
GvHD	Graft-versus-Host Disease
DMFT	Decayed, Missing, and Filled Teeth
ICDAS	International Caries Detection and Assessment System
UWS	Unstimulated Whole Saliva
SWS	Stimulated Whole Saliva
TNF-α	Tumor Necrosis Factor Alpha
HGB	Hemoglobin
HCT	Hematocrit
MCV	Mean Corpuscular Volume
RDW	Red Cell Distribution Width
ESR	Erythrocyte Sedimentation Rate
PLT	Platelet
ANC	Absolute Neutrophil Count
FISH	Fluorescence In Situ Hybridization
R-ISS	Revised International Staging System
PECAM-1	Platelet Endothelial Cell Adhesion Molecule-1
TRIM21	Tripartite Motif-Containing Protein 21
